# Supplementary material for: Melatonin Improves Turbot Oocyte Meiotic Maturation and Antioxidant Capacity, Inhibits Apoptosis-Related Genes mRNAs In Vitro
Source: Antioxidants (Basel). 2023 Jul 6;12(7):1389. doi: 10.3390/antiox12071389 (PMC10376768; doi:10.3390/antiox12071389)
Supplement: Supplementary file 1 [file antioxidants-12-01389-s001.zip › Raw Date.pdf]

Table S1 Gonadosomatic index of turbot during ovarian development (Gonadal weight/body weight).

	Ovary stage	Repeat group 1	Repeat group 2	Repeat group 3	Repeat group 4	Repeat group 5
Body weight		577.03	626.93	774.16	672.3	666.6
Gonadal weight	Prevtg	5.9	5.87	7.24	7.7	6.6
Body weight		1498	1521.3	1688.21	1365.2	1566.6
Gonadal weight	Evtg	39.12	40.21	50.11	39.21	48.2
Body weight		2916.08	1958.25	1926.22	2378.02	3517.75
Gonadal weight	Latvtg	226.5	167.45	181.84	224.55	292.78
Body weight		2184	2053	2435	2666	2872.1
Gonadal weight	Mig-nucle	221.2	239.74	230.2	237.1	281.2
Body weight		2672.62	2687.1	2541.1	2399.3	2890.12
Gonadal weight	Atre	55.17	50.12	48.21	43.2	66.7

Table S2 Proportion of oocyte during ovarian development (Different types of oocyte numbers/Total oocyte number).

Groups	Oocyte stage	Prevtg	Evtg	Latvtg	Mig-nucle	Atre
Repeat group 1	I	174	26	3	3	18
	II	0	35			
	III	0	0	15	2	
	IV	0	0	1	11	
	V	0	0			11
Repeat group 2	I	168	22	3	4	20
	II	0	38			
	III	0	0	18	3	
	IV	0	0	2	10	
	V	0	0			9
Repeat group 3	I	182	21	6	5	15
	II	0	37			
	III	0	0	15	3	
	IV	0	0	3	12	
	V	0	0			13
Repeat group 4	I	191	18	3	3	15
	II	0	31			
	III	0	0	17	3	
	IV	0	0	3	10	
	V	0	0			13
Repeat group 5	I	158	21	5	3	17
	II	0	30			

III	0	0	18	1	
IV	0	0	1	8	
V	0	0			14

Table S3 The GVBD of different diameters of follicle culture in *vitro*.

Repeat group	Control (<0.5mm)	Control (0.5-0.7mm)	Control (>0.7mm)	DHP (<0.5mm)	DHP (0.5-0.7mm)	DHP (>0.7mm)
1	4.2689468	12.25044075	15.82620521	6.639667859	11.10148002	15.83335902
2	5.9676598	9.32382009	11.86267074	4.911360607	12.58636876	19.77559944
3	3.7633934	8.425739164	14.31112405	4.448971534	10.31215121	18.39104155

Table S4 The GVBD of different diameters oocyte culture in *vitro*

Repeat group	Control (<0.5mm)	Control (0.5-0.7mm)	Control (>0.7mm)	DHP (<0.5mm)	DHP (0.5-0.7mm)	DHP (>0.7mm)
1	6.896551724	14.89361702	63.15789474	8.333333333	28.57142857	57.69230769
2	5.263157895	18.91891892	60.97560976	7.317073171	31.11111111	67.39130435
3	6.12244898	14.89361702	74.35897436	9.090909091	30.95238095	75.67567568

Table S5 Effects of melatonin on turbot follicle GVBD (%).

Time	Repeat group	Control	Melatonin (10^{-8}g/L)	Melatonin (10^{-7}g/L)	Melatonin (10^{-6}g/L)	Melatonin (10^{-5}g/L)
	1	0	0	0	0	0
3h	2	0	0	0	0	0
	3	0	0	0	0	0
	1	0	0.100759654	0.683047872	0.839093506	1.226996892
6h	2	0	0.107264503	0.631464336	0.891998509	0.950490711
	3	0	0.114121831	0.716862192	1.168530985	0.829889397
	1	0.179274941	0.231671207	2.415739479	4.768534135	3.01082524
9h	2	0.024396131	0.212609471	1.668181025	3.544151109	4.344452165
	3	0.166560218	0.200011298	2.26070914	3.256637756	4.648490595
	1	0.242390559	0.307299467	6.162589529	12.21956998	13.53593824
12h	2	0.161799434	0.346022042	4.129572987	9.633593495	15.46377698
	3	0.231390007	0.313116455	7.093703576	13.01248278	16.08481734
	1	5.988435571	5.247361285	12.14998797	23.0069338	24.11987457
18h	2	5.238302541	7.223676145	14.07865129	20.69667591	23.09102764
	3	7.547968388	6.041402878	15.51704567	22.86927768	26.99917672
	1	7.727608412	12.29247501	17.34313941	26.27223978	27.04575302
24h	2	10.31549898	10.0803397	19.34131455	27.42302768	30.44983959
	3	12.00075225	9.705515771	16.93304066	29.91867812	31.47839736

Table S6 Effects of melatonin on turbot oocyte GVBD (%).

Time	Repeat group	Control	Melatonin (10^{-8}g/L)	Melatonin (10^{-7}g/L)	Melatonin (10^{-6}g/L)	Melatonin (10^{-5}g/L)
	1	0	0	0	0	0
3h	2	0	0	0	0	0
	3	0	0	0	0	0
	1	0	0.113603464	0.673633561	1.40430908	1.465945662
6h	2	0	0.115279124	0.721542386	1.258438414	1.566840102
	3	0	0.101793613	0.768244054	1.643878506	1.894315536
	1	0.273357798	0.28560915	4.448873916	5.468209188	8.454996306
9h	2	0.340316974	0.26979157	3.623196235	6.310249661	10.07316188
	3	0.445645029	0.304493045	3.827552849	7.217976151	8.403506182
	1	0.311122679	0.399163263	6.757398579	19.95701649	21.44941849
12h	2	0.396473207	0.439366478	7.622378936	17.9680608	19.63857694
	3	0.373347629	0.438261559	9.969968485	16.08198501	18.56558771
	1	9.092154766	7.945070126	16.23862056	28.64840732	26.11715773
18h	2	7.178889401	6.237703768	18.83909981	23.86673373	31.40283654
	3	9.361825979	7.280584177	18.25166982	26.01334139	31.95887693
	1	19.76996208	15.09475426	22.89667125	40.23619537	44.40648388
24h	2	14.63297078	11.98337449	18.75256829	35.88474528	37.08588567
	3	11.90234198	11.74762883	18.98939767	36.95797513	41.53670415

Table S7 Effects of DHP on turbot follicle GVBD (%).

Time	Repeat group	Control	Melatonin (10^{-8}g/L)	Melatonin (10^{-7}g/L)	Melatonin (10^{-6}g/L)	Melatonin (10^{-5}g/L)
	1	0	0	0	0	0
3h	2	0	0	0	0	0
	3	0	0	0	0	0
	1	0	0	0	0	0
6h	2	0	0	0	0	0
	3	0	0	0	0	0
	1	0.109547459	0.131736046	0.11115063	0.135752919	0.157610354
9h	2	0.218073169	0.151421499	0.108167261	0.144106818	0.142876636
	3	0.046350372	0.147073745	0.125026668	0.120367851	0.163484009
	1	0.163681117	0.187603029	0.209584551	0.320840013	0.2662963
12h	2	0.24620092	0.296642814	0.292817558	0.216500523	0.317911091
	3	0.232117962	0.241334157	0.170239184	0.158233108	0.207792609
	1	7.450023442	6.737838448	5.450890228	6.757791929	6.190942815
18h	2	5.078074679	5.074525014	7.622552168	5.215637588	7.737339143
	3	6.43625188	6.962343038	6.029851093	7.401089236	5.636068041
	1	8.809667964	11.7117805	11.80908682	11.70551368	13.38619465
24h	2	12.64727043	8.154252962	8.354048	11.35225536	9.420258345
	3	8.870725502	10.80141434	10.84502832	8.745945071	10.857897

Table S8 Effects of DHP on turbot oocyte GVBD.

Time	Repeat group	Control	Melatonin (10^{-8}g/L)	Melatonin (10^{-7}g/L)	Melatonin (10^{-6}g/L)	Melatonin (10^{-5}g/L)
	1	0	0	0	0	0
3h	2	0	0	0	0	0
	3	0	0	0	0	0
	1	0	0.204347515	0.890018398	0.992409407	1.084245111
6h	2	0	0.240279712	0.619150611	0.998108032	1.65283264
	3	0	0.199226397	0.917781785	0.898682561	1.208199297
	1	0.294484221	0.472642767	2.069178251	4.188962871	3.32065568
9h	2	0.319157801	0.40241143	2.721916	3.141138477	4.527685883
	3	0.156377979	0.433616924	2.549807947	1.76373378	5.459485635
	1	0.412068009	0.476835091	7.400844771	9.793078778	12.98663528
12h	2	0.355769169	0.487155574	6.436776641	11.31819414	12.71819695
	3	0.324024958	0.387026585	6.21039666	11.13269849	12.72156777
	1	9.579196973	7.911692922	13.90516337	19.69984813	24.81114447
18h	2	9.033960642	9.032178579	13.19727212	21.90262421	25.11093666
	3	7.278630411	10.2662801	15.42076451	21.27219433	25.06991886
	1	17.0958562	14.43845721	16.39801853	28.52227312	30.41915347
24h	2	12.67827335	12.09460806	19.42853805	25.56206688	35.12871502
	3	14.49378712	15.78326307	19.59893019	25.84827723	28.85606477

Table S9 The expression of *mtnrs* mRNA.

	Repeat group	Mtnr1	Mtnr2	Mtnr3
Oocyte	1	1.200088874	0.244197016	0.887818289
	2	1.087164879	0.220547603	0.796387642
	3	0.913626377	0.215786288	0.701157158
	4	0.966747845	0.211394328	0.71557
	5	0.867779396	0.168484804	0.679393399
Follicle	1	1.739896288	0.085679335	0.187444476
	2	1.563991609	0.066234491	0.189452047
	3	1.786652315	0.068371601	0.173240218
	4	1.555728196	0.076499611	0.195746975
	5	1.670793513	0.078806526	0.166770618

Table S10 The expression of *mtnrs* mRNA in follicle.

	Repeat group	Mtnr1	Mtnr2	Mtnr3
Control	1	23.15780387	1.173206981	1.391755782
	2	20.25077252	0.921756023	1.655687728
	3	16.66428444	0.924718141	0.945787738
Melatonin (10^{-6}g/L)	1	102.578203	0.916326753	0.744288601
	2	91.07504302	0.720086216	0.720086216
	3	95.87155684	0.671217682	0.671217682

Table S11 Effect of melatonin on *mtnrs* expression in oocyte.

	Repeat group	Mtnr1	Mtnr2	Mtnr3
Control	1	19.55481498	1.077736701	0.354286696
	2	17.50489784	1.046251577	0.487446688
	3	15.99120693	0.886852106	0.593625185
Melatonin (10^{-6}g/L)	1	17.27302933	0.072176284	0.107855436
	2	16.32038269	0.123399912	0.071209792
	3	17.19865708	0.769573875	0.103527839

Table S12 Effect of melatonin on the antioxidant capacity of follicle and oocyte.

		Repeat group	follicle	oocyte
SOD activities	Control	1	3.59231499	4.948542159
		2	4.034304432	2.992898332
		3	4.951820814	4.692089818
	Melatonin (10^{-6}g/L)	1	6.707746087	5.964263047
		2	7.983990431	7.427252447
		3	6.640359128	6.464321565
CAT activities	Control	1	142.0077276	82.56404263
		2	133.884472	97.07547752
		3	101.5647201	91.80404363
	Melatonin (10^{-6}g/L)	1	161.0108528	130.4110095
		2	195.4486038	115.7343045
		3	188.7505486	112.6633752
GSH-Px activities	Control	1	3.915826271	3.739082328
		2	3.14444589	2.298594168
		3	3.349746178	3.568014894
	Melatonin (10^{-6}g/L)	1	9.88141757	6.104591195
		2	8.354639224	6.474015125
		3	7.589518814	5.917320277
GSH content	Control	1	17.64906499	21.59321262
		2	22.76154411	25.58061607
		3	23.35394606	26.76661479
	Melatonin (10^{-6}g/L)	1	38.56493761	31.1003376
		2	50.07854568	39.09275426
		3	52.5487167	41.19382007

MDA content	Control	1	1.435039078	1.490085515
		2	1.206630402	1.296193472
		3	1.423049305	1.461854036
	Melatonin (10^{-6}g/L)	1	0.688397714	0.814528695
		2	0.774424727	0.726515983
		3	0.796033894	0.824792369
MDA content	Control	1	127285.675	72317.34595
		2	125748.0916	65783.47891
		3	119856.3463	65296.48388
	Melatonin (10^{-6}g/L)	1	59662.35117	47139.99783
		2	59351.33117	46782.57906
		3	53900.05189	41239.83773

Table S13 Relative expression of *p53*, *caspae3*, *bax*, *bcl2* mRNAs and bax/bcl2 in follicle.

		Repeat group	follicle	oocyte
p53	Control	1	1.132681946	0.518481475
		2	0.990882495	0.459561015
		3	0.948459694	0.48000968
	Melatonin (10^{-6}g/L)	1	0.33541095	0.518481475
		2	0.282047357	0.459561015
		3	0.327803137	0.48000968
caspase3	Control	1	0.963255805	1.292898167
		2	1.067269735	1.148947544
		3	0.97271177	1.166669734
	Melatonin (10^{-6}g/L)	1	0.121662938	0.033103976
		2	0.114559855	0.037724607
		3	0.128339031	0.015101958
bax	Control	1	0.819296918	0.483620235
		2	1.030095896	0.406863389
		3	1.184898151	0.414227511
	Melatonin (10^{-6}g/L)	1	0.131377089	0.115355125
		2	0.15995842	0.135549038
		3	0.144932972	0.133652456
bcl2	Control	1	1.271956275	1.060269357
		2	0.913020061	0.868802521
		3	0.861087922	0.878413655
	Melatonin (10^{-6}g/L)	1	2.353685848	1.299571367
		2	2.252086289	1.608773137
		3	2.22285182	1.813371313

bax/bcl2	Control	1	0.644123492	0.061577025
		2	1.128229203	0.058335726
		3	1.376047812	0.071960901
	Melatonin (10^{-6}g/L)	1	0.456129597	0.088763978
		2	0.468303647	0.052430361
		3	0.471563151	0.074749742
