# Supplementary material for: Melatonin Improves Turbot Oocyte Meiotic Maturation and Antioxidant Capacity, Inhibits Apoptosis-Related Genes mRNAs In Vitro
Source: Antioxidants (Basel). 2023 Jul 6;12(7):1389. doi: 10.3390/antiox12071389 (PMC10376768; doi:10.3390/antiox12071389)

Figure S1. Proportions of different size of oocytes during isolated samples. Values are represented as the mean ± SEM (n =3). Bars with different superscripts are statistically different ($P < 0.05$)

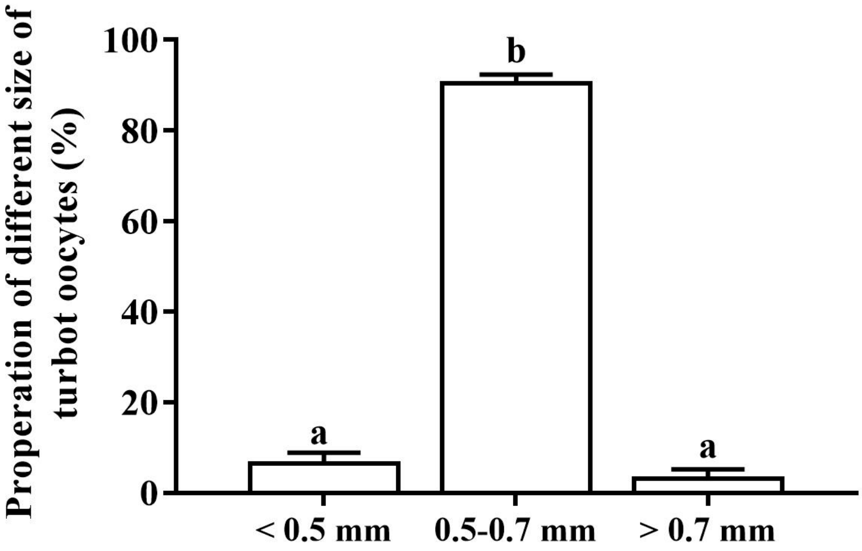

Figure S2. Effect of melatonin on cAMP synthetic (*ac3α*, *ac3β*) and hydrolytic (*pde3α*, *pde3β*) genes expression, cAMP content and maturation-related gene *mPRα* in turbot follicle and oocyte after treatment for 24 hours. Values are represented as the mean ± SEM (n = 9). Bars with different superscripts are statistically different ($P < 0.05$). ac3α: adenylate cyclase 3α; ac3β: adenylate cyclase 3β; pde3α: phosphodiesterase 3α; pde3β: phosphodiesterase 3β; mPRα: membrane progesterone receptor α

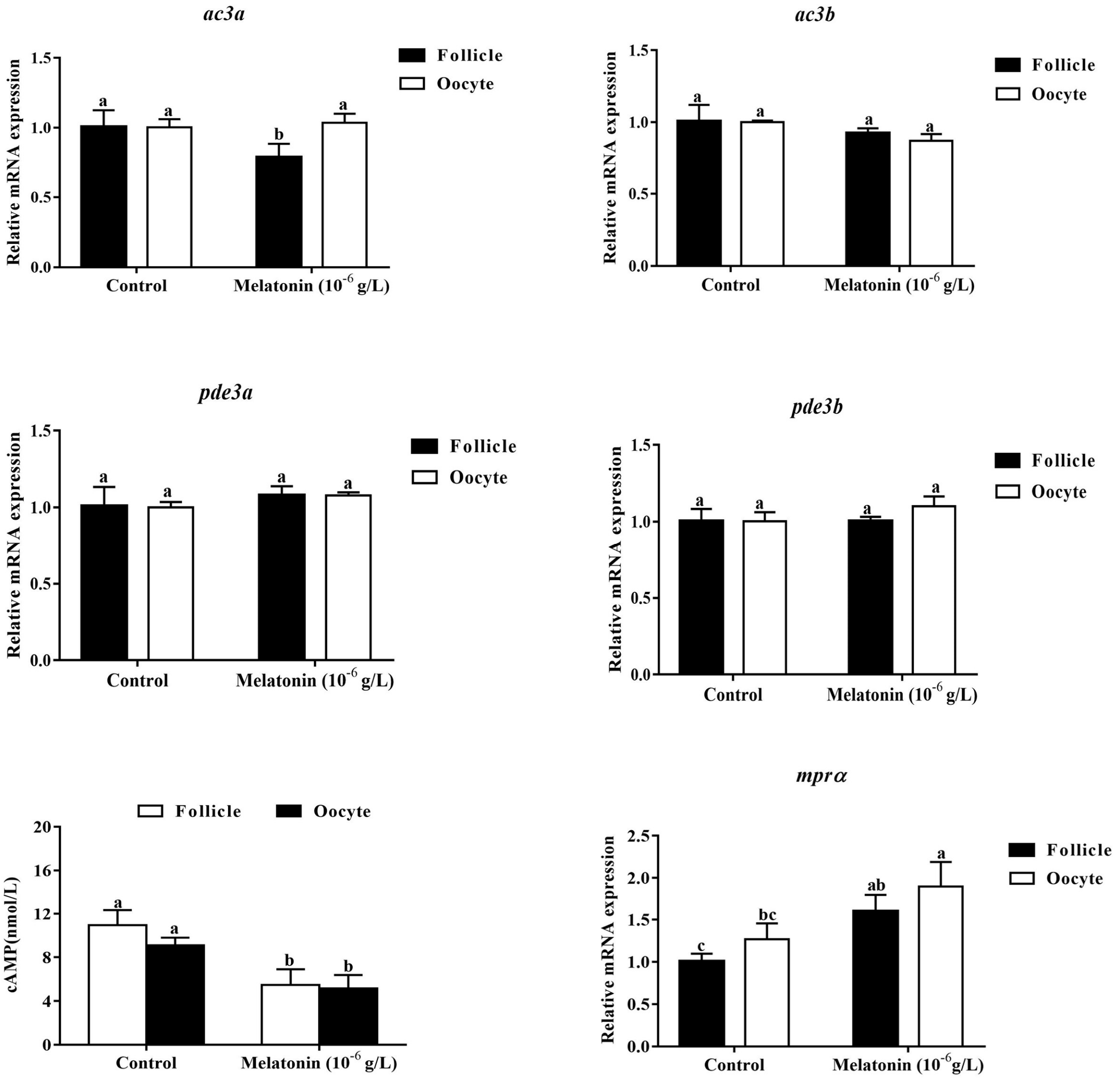

Figure S3. Survival rate of turbot oocyte (A, C) and follicle (B, D) culture in *vitro* treatment with melatonin and DHP. Values are represented as the mean ± SEM (n = 9).

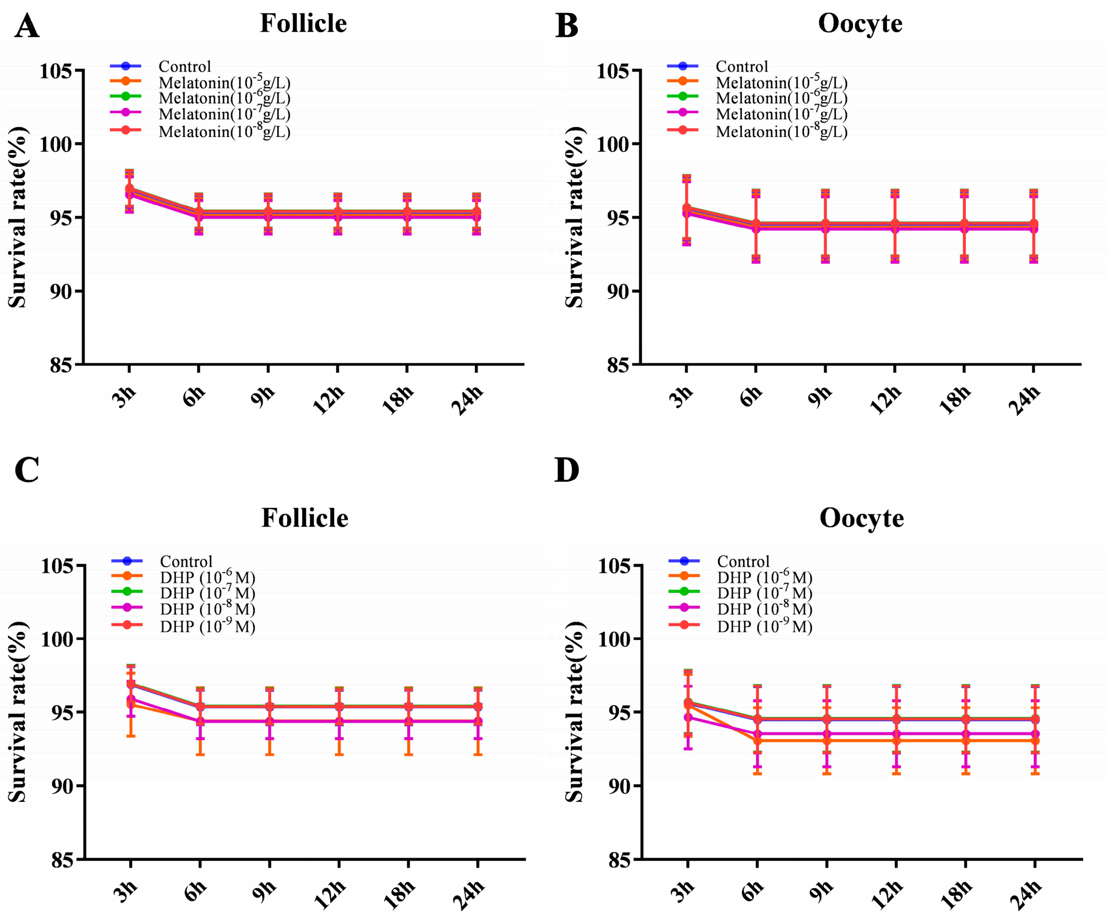

Figure S4. The amplification plot and melt curve of all used genes.

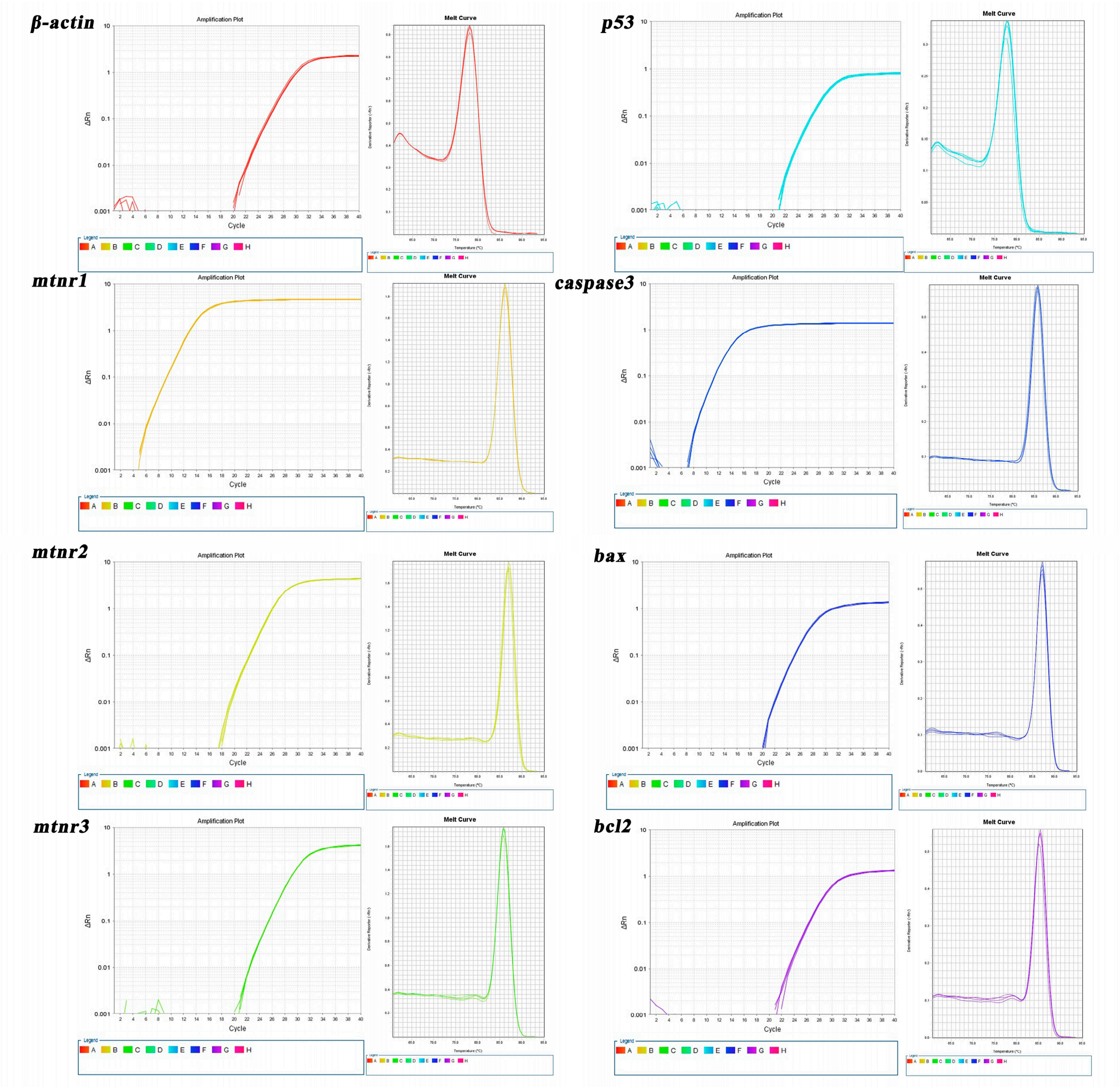

Figure S5. The melatonin levels in medium after treatment for different times. Values are represented as the mean ± SEM (n = 9).

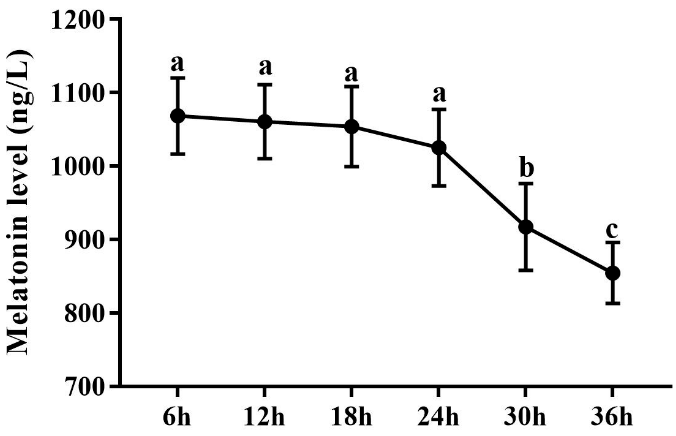

Supplement: Supplementary file 1 [file antioxidants-12-01389-s001.zip › Supplemental data.pdf]
